# Dataset Condensation via Expert Subspace Projection

**DOI:** 10.3390/s23198148

**Published:** 2023-09-28

**Authors:** Zhiheng Ma, Dezheng Gao, Shaolei Yang, Xing Wei, Yihong Gong

**Affiliations:** 1 Shenzhen Institute of Advanced Technology, Chinese Academy of Sciences, Shenzhen 518055, China; zh.ma@siat.ac.cn; 2 Institute of Artificial Intelligence and Robotics, Xi’an Jiaotong University, Xi’an 710049, China; gaodezheng@stu.xjtu.edu.cn (D.G.); ygong@mail.xjtu.edu.cn (Y.G.); 3 School of Software Engineering, Xi’an Jiaotong University, Xi’an 710049, China; yangshaolei@stu.xjtu.edu.cn

**Keywords:** dataset condensation, synthetic data, subspace optimization, deep learning

## Abstract

The rapid growth in dataset sizes in modern deep learning has significantly increased data storage costs. Furthermore, the training and time costs for deep neural networks are generally proportional to the dataset size. Therefore, reducing the dataset size while maintaining model performance is an urgent research problem that needs to be addressed. Dataset condensation is a technique that aims to distill the original dataset into a much smaller synthetic dataset while maintaining downstream training performance on any agnostic neural network. Previous work has demonstrated that matching the training trajectory between the synthetic dataset and the original dataset is more effective than matching the instantaneous gradient, as it incorporates long-range information. Despite the effectiveness of trajectory matching, it suffers from complex gradient unrolling across iterations, which leads to significant memory and computation overhead. To address this issue, this paper proposes a novel approach called Expert Subspace Projection (ESP), which leverages long-range information while avoiding gradient unrolling. Instead of strictly enforcing the synthetic dataset’s training trajectory to mimic that of the real dataset, ESP only constrains it to lie within the subspace spanned by the training trajectory of the real dataset. The memory-saving advantage offered by our method facilitates unbiased training on the complete set of synthetic images and seamless integration with other dataset condensation techniques. Through extensive experiments, we have demonstrated the effectiveness of our approach. Our method outperforms the trajectory matching method on CIFAR10 by 16.7% in the setting of 1 Image/Class, surpassing the previous state-of-the-art method by 3.2%.

## 1. Introduction

With the rapid development of the Internet, a growing number of large-scale datasets are being collected for obtaining state-of-art machine learning models in multiple fields, including computer vision, natural language processing, and speech recognition [1]. Such rapid growth of dataset scale results in increasingly expensive model training, and at some scales, even storing and preprocessing the data are burdensome. For instance, the training of a recent language model, GPT-3 [2], consumes an astonishing 190 MWh of electricity, generating approximately 85,000 kg of CO${}_{2}$, according to US carbon emission standards, which is equivalent to the emissions of driving a car 700,000 km. An intuitive solution is to select a representative subset from the original dataset, commonly referred to as coreset selection. However, previous work shows that, when facing strict compression ratios, coreset selection methods suffer from severe information loss [1] and cannot compete with dataset condensation techniques [3,4], which distill the original dataset into a much smaller synthetic dataset. Typically, this type of method adopts bi-level optimization, which involves an inner optimization for model updates and an outer optimization for synthetic image updates. To distill the essential information from the original dataset into the synthetic dataset, a suitable matching objective must be defined.

The matching objective includes distribution matching [5], gradient matching [6,7], and meta-model matching [8,9]. Notably, a trajectory matching objective [10] has recently been introduced, demonstrating significant performance improvements over other matching objectives. This objective aims to align the trajectory of the network trained with the synthetic dataset images with the parameter trajectory of the network trained with the original dataset. Unlike gradient matching methods, which only consider the instantaneous training dynamics (i.e., gradients) between the synthetic and real datasets, trajectory matching recognizes that the long-range training dynamics (i.e., training trajectory) provide more informative constraints for dataset condensation.

However, the limitation of trajectory matching is the substantial cost of computation and memory involved in executing multiple unrolled gradient computations for the recursive computational graph when mimicking the training trajectory. This hampers the feasibility of training on the complete set of synthetic images without resorting to slicing and may introduce bias when conducting mini-batch training on the sliced data, ultimately affecting the final performance.

**Figure 1:**
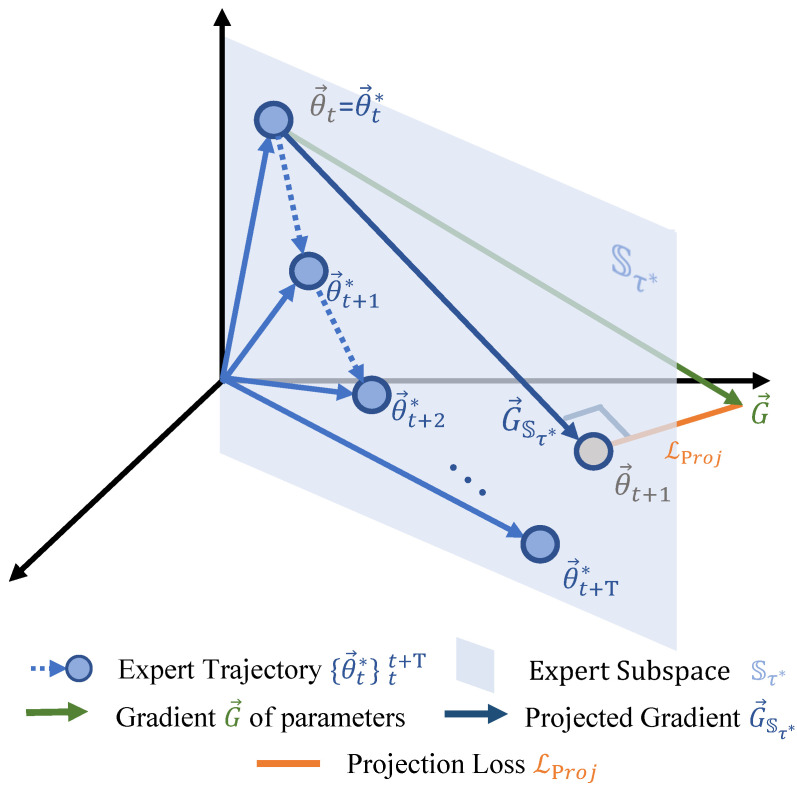
Diagram illustrating the proposed Expert Subspace Projection (ESP) method. The expert trajectory consists of weight snapshots obtained during training with the original dataset $D_{real}$. Each node ${\vec{\theta }}_{t}^{*}$ represents a saved weight snapshot at the end of step *t*. The subspace $S_{\tau^{*}}$ is spanned by the set of weight snapshots ${\left\{{\vec{\theta }}_{t}^{*}\right\}}_{t}^{t+T}$. $\vec{G}$ represents the parameter gradient vector generated during training with the synthetic dataset $D_{syn}$. The subspace projection loss $L_{Proj}$ penalizes the norm of the residual vector between $\vec{G}$ and its projection ${\vec{G}}_{S_{\tau^{*}}}$ within the subspace $S_{\tau^{*}}$.

This paper presents a novel matching objective called Expert Subspace Projection (ESP), which effectively guides the dataset condensation process with long-range training dynamics while significantly reducing computational and memory costs compared to trajectory matching. Instead of strictly enforcing the synthetic dataset’s training trajectory to mimic the real dataset, ESP only constrains it to lie within the subspace spanned by the training trajectory of the real dataset. Our core technical concept is illustrated in Figure 1. We extract an arbitrary segment from the parameter trajectory obtained through training on the original dataset $D_{real}$, whose starting point is ${\vec{\theta }}_{t}^{*}$ and ending point is ${\vec{\theta }}_{t\;+\;T}^{*}$. For training with the synthetic data, the neural network is initialized with ${\vec{\theta }}_{t}^{*}$, and the gradient of the parameters at that point is $\vec{G}$. In gradient matching [6], the objective is to align $\vec{G}$ with ${\vec{\theta }}_{t\;+\;1}^{*}\;-\;{\vec{\theta }}_{t}^{*}$. Conversely, in trajectory matching [10], the objective is to align ${\vec{\theta }}_{t\;+\;T}$ with ${\vec{\theta }}_{t\;+\;T}^{*}$ after *T* iterations. Both methods have their advantages and disadvantages. Gradient matching, although computationally simpler, cannot effectively utilize long-range information. On the other hand, trajectory matching has the ability to incorporate long-range information but requires the gradient to be unrolled through *T* iterations during computation.

Our method, however, circumvents the drawbacks of both approaches while inheriting their respective advantages. First, we construct a subspace $S_{\tau^{*}}$ span by means of the training trajectory ${\left\{{\vec{\theta }}_{t}^{*}\right\}}_{t}^{t\;+\;T}$. This subspace effectively captures a substantial amount of information relevant to the training trajectory using a real dataset. Consequently, by confining the optimization gradient $\vec{G}$ of each step within this subspace when training with the synthetic dataset, we are able to distill a significant portion of the information inherent in the real dataset into the synthetic one. Specifically, we define a new objective function $L_{Proj}$ to penalize the norm of the residual vector between $\vec{G}$ and its projection ${\vec{G}}_{S_{\tau^{*}}}$ within the subspace $S_{\tau^{*}}$. This approach not only circumvents the requirement for gradient unrolling across *T* iterations but also effectively utilizes the long-range information embedded within the optimization process. The memory consumption of our method is not affected by the number of steps in the neural network optimization (inner optimization) since the synthetic images are updated at every step. This significantly reduces the spatial complexity of training compared to previous trajectory methods. Consequently, our method facilitates training on the entire set of synthetic images without the need for slicing and can be seamlessly integrated with other techniques such as distribution matching [5] and KFS [1]. Our method has been extensively tested on four widely used data condensation benchmarks, and the results demonstrate its remarkable effectiveness. In particular, our ESP method outperforms trajectory matching by a significant margin, leading to the establishment of a new state-of-the-art performance.

## 2. Related Work

### 2.1. Dataset Condensation

In order to reduce the resources required for deep neural networks, researchers usually use knowledge distillation [11,12,13,14] to distill complex and large models into smaller ones, while still ensuring comparable results to those before compression. As technology has evolved, the concept of knowledge distillation began to be transferred to the datasets. Wang et al. [4] first proposed dataset condensation, which uses meta-learning [8,9] methods to compress the knowledge of the entire training dataset into a small amount of synthetic data and achieves high accuracy through several steps of gradient descent on the synthetic data. Subsequently, many works have utilized gradient matching [6,7,10] and distribution matching [1,5] for optimization. Dataset Condensation (DC) [6] assumes that the optimization process of the synthetic dataset is very close to the real dataset, so it optimizes the synthetic dataset by matching the optimization trajectory of the model trained on the synthetic dataset with the optimization trajectory of the real dataset. Differentiable Siamese Augmentation (DSA) [7] is further work based on DC [6] and uses a set of data enhancement strategies while learning the synthetic image, thereby enhancing the information in the real training image and transferring this enhanced knowledge to the synthetic image. Distribution matching (DM) [5] matches the features of the real samples and the synthetic samples that are output at the last layer of the neural network. These neural networks are randomly initialized to ensure computational efficiency and, at the same time, very high accuracy. Knowledge Factorization and Sharing (KFS) [1] also uses distribution matching [5], introducing a new latent code decoder architecture, which greatly increases the number of modalities of synthetic images with the same number of parameters, thus achieving a new state of the art.

Trajectory matching [10] encourages the synthetic dataset to mimic the long-range training dynamic of the real dataset by mimicking the expert trajectory generated by the real dataset. Although long-range information helps it achieves satisfactory results, the method requires accumulating multiple computational graphs, which greatly increases memory consumption and even introduces a subsampling bias as a result of having to reduce the batch size for saving memory. Our ESP method alleviates the memory consumption issue by projecting the model gradient into expert subspace instead of matching model parameters, allowing the model to be trained on the complete synthetic dataset while also utilizing the long-range information of expert trajectories.

### 2.2. Subspace Training

Generally, deep neural networks come with a large number of parameters, which tend to have strong correlations, thus resulting in great redundancy. Guy et al. [15] first proposed the hypothesis that, in various large-scale deep learning scenarios, gradients dynamically converge to a very small subspace after short-term training, so gradient descent in the subspace will yield a similar loss reduction. Li et al. [16] try to optimize network parameters in a small, random subspace instead of the original parameter space, then slowly increase the dimension of this subspace. Eventually, the authors find that the intrinsic dimension required for model training is smaller than one might think. While this training holds promise for more efficient and scalable optimization schemes, its practical application is limited by poor optimization performance. Gressmann et al. [17] made some optimizations to the stochastic subspace approach, achieving further improvements by applying independent projections to different parts of the network, making the approximation more efficient as the network dimensionality grows. After that, Li et al. [18] extracted the landscape by analyzing the optimization trajectories, while also verifying that many standard neural network structures can be trained well with only 40 independent variables and that the performance is almost the same as conventional training with all parameters. Inspired by [18], we span the parameters from the expert trajectory into an expert subspace and encourage the synthetic data to learn information about real data within the expert subspace, thereby optimizing synthetic data in an efficient way.

### 2.3. Coreset Selection

Coreset selection [19,20,21,22,23] is an approximate replacement of the original large dataset with a small dataset such that the small dataset still provides rich information, making the accuracy on the test dataset very close to the original dataset. However, such methods often come with a trade-off between performance and dataset size, as they produce a rough approximation of the full dataset. Dataset condensation is very similar to coreset selection, but dataset condensation [1,5,6,10] is more robust. It mainly uses the original dataset to synthesize some learnable pictures and then captures the rich information encoded in the original dataset to realize the compression of the original dataset. These learned images do not appear in the original dataset.

## 3. Method

The primary objective of dataset condensation is to efficiently compress a real dataset $D_{real}$ into a significantly smaller synthetic dataset $D_{syn}$ while minimizing the loss in performance during downstream training. In line with previous studies [6,7,10], our methods also embrace a bi-level optimization framework. This framework consists of an inner optimization for the neural network’s parameters and an outer optimization for synthetic images.

**Figure 2:**
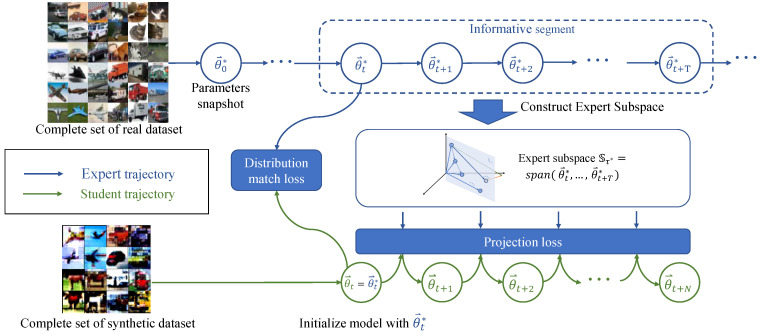
Overview of our ESP method for dataset condensation. We train the model with the complete real dataset to obtain the expert trajectory. An informative segment of length *T* is extracted and flattened into one-dimensional vectors, forming a subspace. The condensation process utilizes a bi-level optimization framework. The inner stage refines the model’s parameters within the expert subspace using projected gradient descent, while the outer stage updates the synthetic dataset. Distribution matching loss penalizes feature distribution discrepancy, and projection loss penalizes gradients outside the expert subspace.

To begin with, we present the comprehensive framework of our Expert Subspace Projection (ESP) method, as illustrated in Figure 2. In the inner optimization, we utilize the binary cross-entropy (BCE) loss as the objective function. This loss penalizes the misclassification of synthetic images based on their pre-defined ground-truth labels. Notably, rather than using the original gradient descent, we update the neural network’s parameters using projected gradient descent within the expert subspace. This constraint ensures that the training trajectory (student trajectory) remains confined within the expert subspace. In the outer optimization, we employ a combination of the projection loss and the distribution matching loss [5] as the objective function. The first loss term penalizes any deviation of gradients from the desired subspace, while the second term aligns the feature distribution between real and synthetic images. During this process, the synthetic images are updated using normal gradient descent.

In subsequent sections, we delve into the technical details of our method, providing a comprehensive explanation of its underlying mechanisms. Furthermore, we conduct a thorough analysis to demonstrate the memory consumption advantage offered by our approach.

### 3.1. Preliminaries

The long-range training trajectory of the original dataset, referred to as the expert trajectory, has been empirically demonstrated to be effective in guiding the condensation of the synthetic dataset in trajectory matching [10]. As illustrated in Figure 2, an expert trajectory $\tau^{*}={\left\{{\vec{\theta }}_{t}^{*}\right\}}_{t}^{t\;+\;T}$ consists of a series of parameter snapshots during the training process with $D_{real}$. Each snapshot, denoted as ${\vec{\theta }}_{t}^{*}$, represents the parameters saved after the *t*-th epoch and is flattened into a one-dimensional vector. In the context of trajectory matching, the inner optimization process involves *T* steps of updates on the neural network’s parameters, i.e., from ${\vec{\theta }}_{t}$ to ${\vec{\theta }}_{t\;+\;T}$, supervised by the classification loss of the synthetic images $D_{syn}$. To ensure that the student trajectory begins from the same starting point as the expert trajectory, ${\vec{\theta }}_{t}$ is initialized with ${\vec{\theta }}_{t}^{*}$. The outer optimization process involves a single update step for the synthetic images, supervised by the trajectory matching loss:

(1) $$L_{TM}=\frac{∥{\vec{\theta }}_{t\;+\;N}-{\vec{\theta }}_{t\;+\;T}^{*}{∥}_{2}^{2}}{∥{\vec{\theta }}_{t}^{*}-{\vec{\theta }}_{t\;+\;T}^{*}{∥}_{2}^{2}},$$

where *N* is the length of the student trajectory, and *T* is the length of the expert trajectory. It is worth noting that the student trajectory’s length is not necessarily the same as that of the expert trajectory due to the much smaller size of the synthetic dataset compared to the original dataset. In practice, *N* is intentionally set to be smaller than *T*.

In most cases, the student trajectory is indeed much shorter. However, for the sake of simplicity and without loss of generality, we assume that both trajectories have the same length in the equation provided above. $L_{TM}$ promotes $D_{syn}$ to mimic the long-range training dynamic of $D_{real}$. However, due to the inclusion of *T* steps of updates in the inner optimization process, the calculation of gradients for the outer optimization requires unrolling the gradients through *T* iterations. This unrolling process and the need to save the computation graph from multiple updates result in significantly higher memory consumption compared to other methods [5,6,7]. This high memory usage necessitates the use of a slice of the synthetic dataset, which may introduce a biased optimization. In Section 3.5, we discuss the impact of this issue in detail and analyze the memory usage.

### 3.2. Expert Subspace Projection

Our proposed ESP method leverages the benefits of long-range training dynamics obtained from expert trajectories. Importantly, it addresses the issue of linear memory growth that arises when unrolling the gradient through iterations, as the inner optimization solely entails a single-step update. The crux of the challenge lies in emulating the long-range training dynamics despite the constraint of having only one update.

Our solution is simple yet effective. In our approach, we confine each optimization step to remain within the subspace spanned by the expert trajectory during the inner optimization. Simultaneously, during the outer optimization, we penalize the residual gradient that deviates from this subspace. The expert subspace $S_{\tau^{*}}$ is spanned by the vector set ${\left\{{\vec{\theta }}_{t}^{*}\right\}}_{t}^{t\;+\;T}$:

(2) $$S_{\tau^{*}}=\mathrm{span}\left({\vec{\theta }}_{t}^{*},\cdots ,{\vec{\theta }}_{t\;+\;T}^{*}\right).$$

By using Schmidt’s orthogonal standardization, we can obtain a set of standardized bases for the subspace $S_{\tau^{*}}$:

(3) $$\begin{array}{cc} E & =\left[\vec{\epsilon_{0}},\vec{\epsilon_{1}},\cdots ,\vec{\epsilon_{T}}\right]\\ \; & =OS(\left[{\vec{\theta }}_{t}^{*},\cdots ,{\vec{\theta }}_{t\;+\;T}^{*}\right]), \end{array}$$

where the notation OS(·) represents the orthogonal standardization operator. It is worth emphasizing that the length of the expert trajectory is considerably smaller than the dimension of the parameter vector. Our experimental findings provide evidence that this expert subspace encapsulates the majority of long-range training dynamics exhibited by the expert trajectories. Therefore, it can serve as a reliable proxy for capturing these dynamics. Consistent with trajectory matching [10], the expert trajectory can be generated and stored offline prior to commencing the data condensation process. This approach helps to conserve memory and reduce computation costs.

### 3.3. Inner Optimization

At the onset of the dataset condensation process, we initialize the synthetic images with random Gaussian distribution, along with pre-assigned category labels. The neural network is initialized with the starting point of the expert trajectory, i.e., ${\vec{\theta }}_{t}:={\vec{\theta }}_{t}^{*}$.

Following the bi-level optimization framework, we commence by updating the neural network in the inner optimization phase, followed by updating the synthetic images in the outer optimization phase. The gradient for the neural network in the inner optimization is computed as follows:

(4) $$\vec{G}=\nabla_{{\vec{\theta }}_{t}}L_{BCE}\left(M\left(D_{syn};{\vec{\theta }}_{t}\right)\right),$$

where M represents the neural network model, and $L_{BCE}$ represents binary cross-entropy loss. Rather than directly utilizing this gradient to update the neural network, we project it onto the expert subspace to align with the long-range training dynamics of the original data:

(5) $${\vec{G}}_{S_{\tau^{*}}}={\mathrm{Proj}}_{S_{\tau^{*}}}\left(\vec{G}\right)=EE^{T}\vec{G},$$

where $E^{T}$ represent the transposition of E, and the neural network is updated by the projected gradient in expert subspace:

(6) $${\vec{\theta }}_{t+1}={\vec{\theta }}_{t}-\alpha {\vec{G}}_{S_{\tau^{*}}},$$

where α represents the learning rate. In the equation, we have introduced the naive gradient descent method. However, it is essential to emphasize that there are other gradient-based optimization algorithms that can also be employed in this context.

### 3.4. Outer Optimization

In the outer optimization, we perceive the gradient deviation outside the expert subspace as the disparity between the synthetic dataset and the original dataset. To alleviate this dissimilarity, we introduce a penalization function that aims to minimize the norm of the residual gradient:

(7) $$L_{Proj}={∥\vec{G}-{\vec{G}}_{S_{\tau^{*}}}∥}_{1},$$

(8) $$L_{DM}=\frac{1}{C}\sum_{c=1}^{C}{∥M\left(D_{real};{\vec{\theta }}_{t}\right)-M\left(D_{syn};{\vec{\theta }}_{t}\right)∥}_{2}^{2},$$

where $∥\cdot ∥$ indicates the $\ell_{2}$ norm. In subsequent experimental analyses, we observed that the integration of this loss function resulted in improved accuracy in downstream training and enhanced the level of detail in synthesized images. These findings provide compelling evidence for the complementary nature of these two loss functions. The final objective function is defined as follows, where β is the hyper-parameter to balance these two losses:

(9) $$L_{Out}=L_{DM}+\beta L_{Proj}.$$

Finally, we can update the synthetic dataset with gradient descent:

(10) $$D_{syn}=D_{syn}-\alpha \nabla_{D_{syn}}L_{Out}.$$

Our comprehensive data condensation process is summarized in Algorithm 1.

### 3.5. Memory Consumption

Trajectory matching [10] involves *N* update steps in the inner optimization and a single update step in the outer optimization. Therefore, the calculation of the gradient for the outer optimization needs to unroll the gradient through all *N* steps. As a result, all intermediate computational graphs and variables must be stored in GPU memory. Accurately estimating GPU memory consumption, also known as the memory footprint, is a complex task that depends on various factors, such as the specific operators utilized in the neural network, the connectivity of the computational graph, and the choice of deep learning framework. However, in theory, the memory footprint scales approximately linearly with the number of inner optimization steps *N*. Our method involves a single step of inner optimization followed by a single step of outer optimization. Therefore, our memory consumption is significantly smaller compared to trajectory matching, while still allowing us to benefit from the valuable long-range expert trajectory.
**Algorithm 1:** Expert Subspace Projection
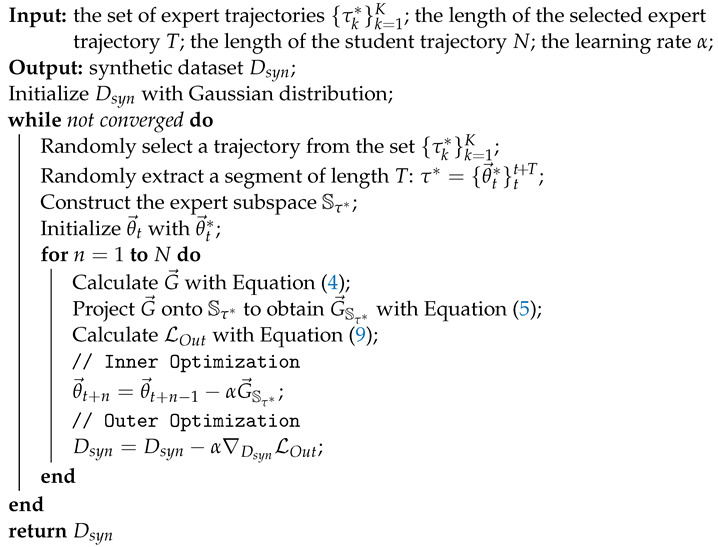


## 4. Experiments

We validate the effectiveness of our proposed method on various classification benchmark datasets, assessing its ability to generalize across different architectures. In addition, we conduct ablation experiments and provide visualization results to further substantiate the efficacy of our approach.

### 4.1. Datasets

**CIFAR10/100 [24]**. The CIFAR10 dataset is a collection of 60,000 color images, each measuring 32 × 32 pixels. These images are classified into 10 distinct categories representing common objects and animals such as airplanes, automobiles, birds, cats, deer, dogs, frogs, horses, ships, and trucks. Each category contains 6000 images, and the dataset is split into a training set with 50,000 images and a test set with 10,000 images. CIFAR10 is widely recognized as a benchmark for image classification tasks, playing a crucial role in the development and evaluation of various machine learning and deep learning algorithms.

CIFAR100 consists of 100 classes, with each class containing 600 images. These 100 classes are grouped into 20 superclasses, with each superclass containing 5 classes. The CIFAR100 dataset covers a diverse range of object categories, including animals, vehicles, household items, and natural objects. Similar to CIFAR10, CIFAR100 is divided into a training set (50,000 images) and a test set (10,000 images). It serves as a benchmark for addressing more intricate and fine-grained classification tasks, enabling researchers to delve into complex image recognition problems.

**TinyImageNet [25]**. TinyImageNet is extensively utilized in computer vision research and benchmarking as a downsized variant of the renowned ImageNet dataset [26]. Its purpose is to offer a more manageable alternative for training and evaluating deep learning models, given the vast scale of the original ImageNet dataset. Comprising 200 distinctive object classes, the TinyImageNet dataset encompasses an approximate quantity of 500 training images, 50 validation images, and 50 test images per class. It boasts a diverse array of object categories, encompassing animals, commonplace objects, as well as a variety of natural and human-created items. Notably, each image within the dataset maintains a resolution of 64 × 64 pixels.

**SVHN [27]**, The SVHN (Street View House Number) Dataset is a valuable collection of real-world images extracted from door numbers captured by Google Street View. It provides a significant amount of data, with more than 70,000 numbers specifically designated for training and an additional 20,000 numbers reserved for testing. Similar to the well-known MNIST [28] dataset, each image in SVHN is 32 × 32 pixels in size and focuses on a single character situated at the center. Notably, many images in SVHN also contain distractors placed alongside the main character of interest. This dataset is highly useful for tasks like digit recognition and character segmentation, allowing researchers to tackle challenges posed by real-world scenarios and evaluate the performance of various machine learning algorithms.

### 4.2. Implementation Details

For fairness and convenience of comparison, we use the same suite of differentiable augmentations as in previous work, as well as the same number of latent code decoder parameters as in [1], which matches the size of 1, 10, and 50 images per class. Prior to the condensation step, we pre-compute 1000 expert trajectories on ConvNet-3 for each dataset. These pre-computed trajectories are then utilized in all our experiments to expedite the condensation process. This approach follows a similar technique used in trajectory matching [10]. During the distillation process, we have the flexibility to randomly select one expert trajectory as the teacher, which saves time in optimizing the expert network.

For the distillation process, we use $\beta =1\times 10^{-5}$ to balance the loss of $L_{DM}$ and $L_{Proj}$. The learning rate of the neural network (ConvNet-3 by default) is set as a constant value of 0.1. The synthetic dataset, represented by the latent code and decoder, follows a linear decay schedule for their learning rates. The initial learning rate for the latent code is set to 0.1, while the initial learning rate for the decoder is set to 0.01. To optimize the model, we employ the SGD as the inner optimizer and Adam [29] as the outer optimizer. More details can be found in Algorithm 1.

For the evaluation, we train the condensed data on ConvNet-3 for accuracy, while using ResNet [30] and DenseNet [31] for cross-architecture ability. To evaluate the performance of classification models trained with the condensed datasets, we report the mean classification accuracy and its corresponding standard deviation across 5 runs with different random seeds.

### 4.3. Comparison with State-of-the-Art Methods

**Figure 3:**
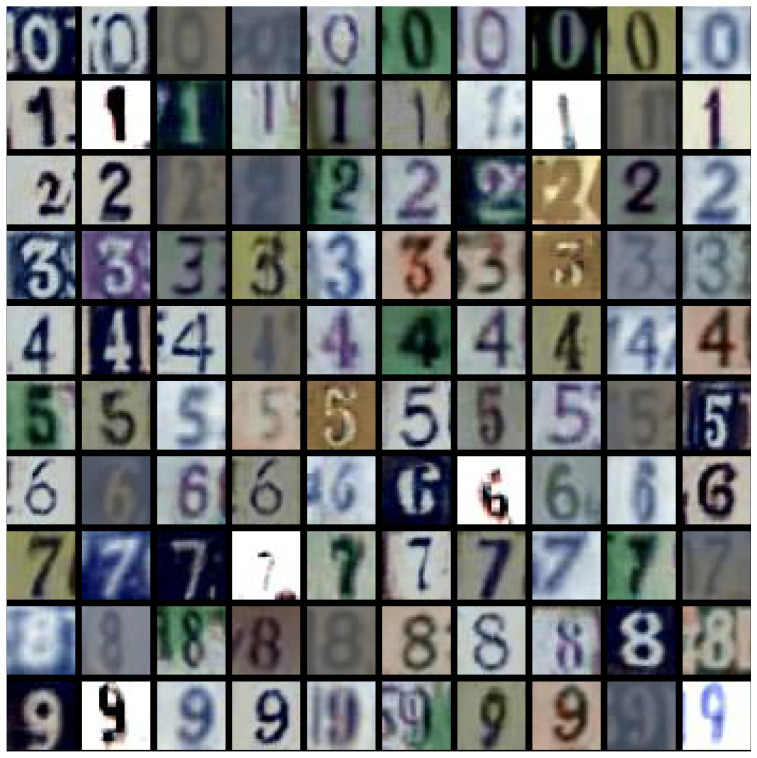
The synthetic images of SVHN [27].

**Figure 4:**
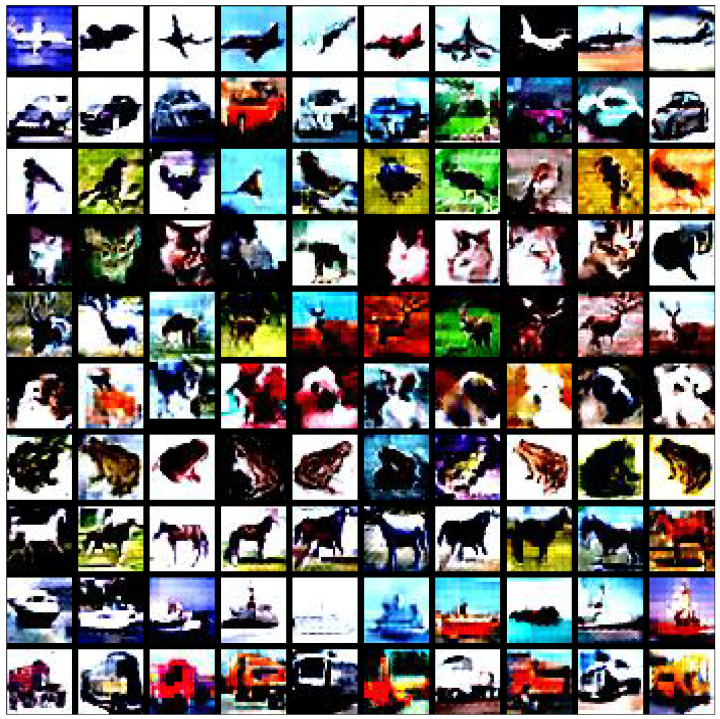
The synthetic images of CIFAR10 [24].

**Figure 5:**
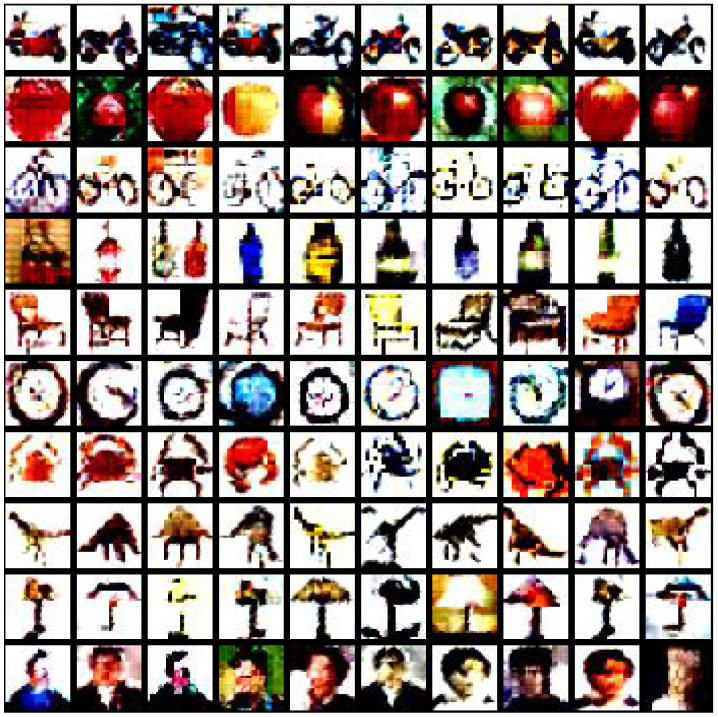
The synthetic images of CIFAR100 [24].

**Figure 6:**
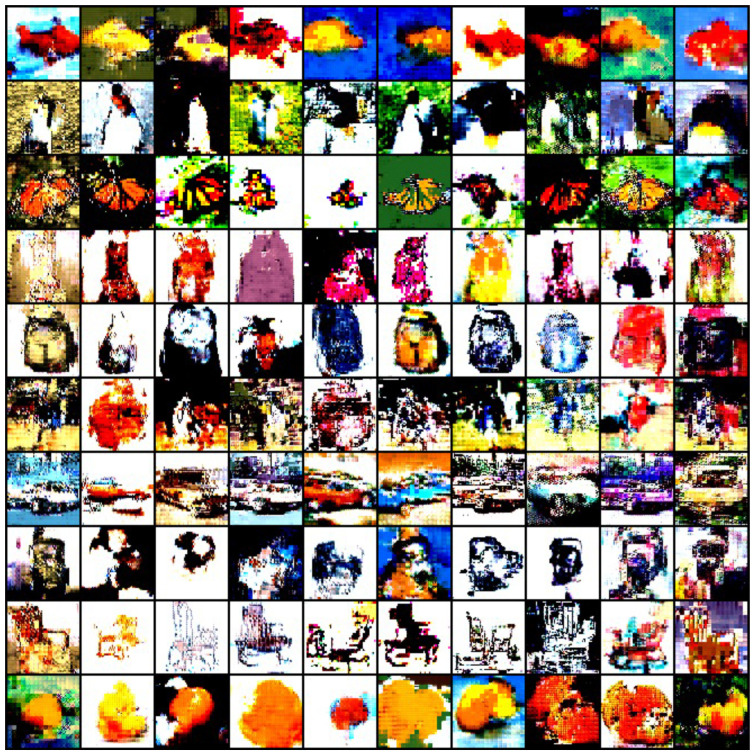
The synthetic images of TinyImageNet [25].

We first compare our ESP method with coreset selection [19,20,21,22,23] methods, such as randomizing [32,33] and herding [34,35] methods. Secondly, we also compare our method with some recent condensation works, namely DC (dataset condensation) [6], DSA (differentiable Siamese augmentation for DC) [7], DM (distribution matching) [5], KIP to NN (infinitely wide convolutional networks) [36], CAFE (aligning features) [37], MTT (matching training trajectories) [10], IDC [38], and KFS (latent space knowledge factorization and sharing) [1]. Our ESP method achieves the state of the art on SVHN [27], CIFAR10 [24], CIFAR100 [24], and TinyImageNet [25] datasets. Table 1 illustrates the performance of our method and competitor methods on four datasets. Particularly when IPC = 1, our ESP method achieves a significant improvement under the same parameters on all four datasets. In particular, the improvements on SVHN, CIFAR10, CIFAR100, and TinyImageNet are 2.3%, 5.4%, 2.3%, and 9.7%, respectively, over other methods, indicating that the subspace projection method improves the data efficiency of dataset condensation. In order to illustrate the effect of our condensed dataset, we visualized our synthetic image of SVHN in Figure 3, CIFAR10 in Figure 4, CIFAR100 in Figure 5, and TinyImageNet in Figure 6.

### 4.4. Cross-Architecture Generalization

In the context of dataset condensation, it is imperative that an ideal synthetic dataset exhibit similar training effects as the original dataset on downstream models with arbitrary structures. Consequently, the performance of cross-architecture generalization holds significant importance as a key metric. We used our synthetic data generated on ConvNet-3 (0.32 M parameters) to train different models, including ResNet-10 [30] (4.90 M parameters), DenseNet-121 [31] (6.96 M parameters), and EfficientNet-V2-s [39] (22 M parameters), and the results are presented in Table 2 and Table 3.

We can see that the performance of ESP is more robust to the change in network architectures and achieves state-of-the-art performance on most of the network architectures. For the SVHN [27] dataset, the accuracy on ResNet-10 is generally higher than our baseline model (ConvNet-3), up to 2.1%. These experiments provide evidence that the synthetic dataset generated by ESP exhibits better generalizability compared to other datasets, showcasing the superior ability of our ESP method to capture representative information from the original datasets. However, the experimental results also reveal that when there is a significant architectural difference between the training and testing phases, the cross-architecture performance is weakened. This suggests that a cross-architecture generalization problem persists across all dataset condensation methods.

### 4.5. Memory Analysis

**Figure 7:**
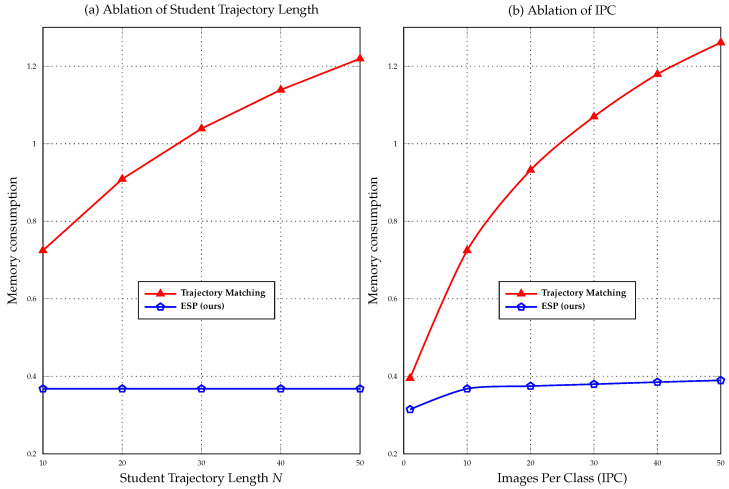
Memory consumption of trajectory matching [10] and our method. The right part shows the memory consumption of different synthetic steps for IPC = 10, and the left part shows the memory consumption of the two methods with different IPC for the CIFAR10 dataset. The results of trajectory matching are obtained by executing their officially released code [10].

We perform an experimental comparison of the memory consumption between our ESP method and trajectory matching [10] on the CIFAR10 [24] dataset. In line with our analysis in Section 3.5, trajectory matching exhibits a linear increase in memory consumption with the length of the student trajectory due to its inner optimization steps being equal to the student trajectory length. This theoretical analysis is confirmed by Figure 7a. Notably, the memory consumption of trajectory matching increases approximately linearly with the length of the student trajectory, while our ESP method remains unaffected.

We conducted an additional experiment to demonstrate that our ESP method has a significantly lower growth rate in memory consumption compared to trajectory matching when increasing the size of the synthetic dataset. Figure 7b illustrates this observation, where our ESP method only exhibits a slight increase in memory usage as the IPC increases. In contrast, the memory consumption of the trajectory matching [10] method experiences a substantial surge, rendering it ineffective for training on the complete synthetic dataset with higher IPC. Conversely, our method can directly handle the complete synthetic dataset, enabling unbiased training, as we elaborate on in Section 4.6.

### 4.6. Synthetic Batch Size Analysis

**Figure 8:**
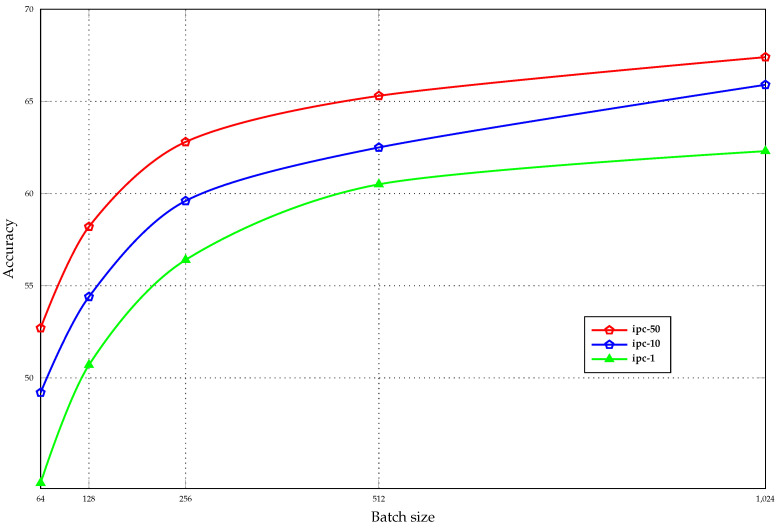
Accuracy on ConvNet-3 of synthetic images trained with different batch sizes.

Based on the analysis presented in [1], it is noteworthy that training the synthetic dataset using batch optimization, where the complete synthetic dataset is divided into several batches, introduces a bias in the gradient. This bias stems from disregarding the interactions between different synthetic images, resulting in a reduction in diversity among images within classes. The ablation experiments shown in Figure 8 quantify the impact of this issue. In the figure, we trained synthetic images on the CIFAR10 dataset with various batch sizes, ranging from small (batch size = 64) to large (batch size = 1024). It is evident that the accuracy increases with batch size and stabilizes with larger batch sizes, reflecting the property that the bias of the gradient of the synthetic dataset decreases as the batch size increases. Thanks to the efficient use of memory in our ESP method, we are able to train on the complete set of the synthetic dataset, which allows us to achieve better performance than trajectory matching.

### 4.7. Ablation Study

**Figure 9:**
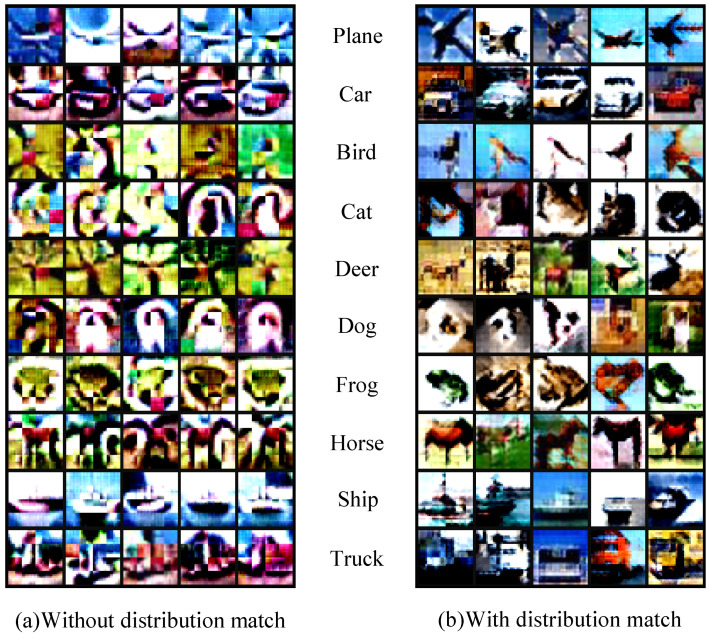
The condensed synthetic images produced by our method exhibit a noticeable difference when comparing the left and right parts. The left portion corresponds to images trained without the distribution loss, resulting in a more abstract style. On the other hand, the right portion showcases images with more pronounced and detailed texture information. Experiments are conducted on CIFAR10 [24].

As discussed in Section 3.4, the projection loss and the distribution matching loss exhibit a high level of complementarity. The projection loss focuses on aligning the long-range training dynamics between the synthetic and original datasets, while the distribution matching loss aims to match the static feature distribution between the two datasets. These two loss components work together to ensure a comprehensive alignment of both the dynamic and static aspects of the datasets. This observation is further supported by the results of our ablation experiments, as presented in Table 4. The individual losses alone show suboptimal performance, whereas their combination yields excellent results. We further visualize the synthetic images with and without the distribution loss. As can be observed in Figure 9, the introduction of the distribution loss results in synthetic images that exhibit more detailed textures and recognizable visual concepts. This phenomenon may be attributed to the fact that the projection loss primarily constrains higher-order information, such as gradients. On the other hand, low-level information like texture is predominantly constrained by the distribution matching loss.

## 5. Conclusions

In this paper, we have proposed a novel dataset condensation method called Expert Subspace Projection (ESP) that effectively utilizes long-range training dynamics while reducing computational overhead compared to prior trajectory matching techniques. Our key insight is to constrain model optimization to remain within the subspace spanned by expert trajectories from the original dataset. This avoids expensive unrolling of gradients across multiple steps, enabling memory-efficient training of the complete set of synthetic data. We have validated ESP extensively on image classification tasks, demonstrating state-of-the-art results on CIFAR, SVHN, and TinyImageNet datasets compared to existing condensation methods. Importantly, we have shown ESP’s superior ability to transfer condensed datasets to unseen architectures, indicating it effectively distills dataset knowledge in an architecture-agnostic manner. Overall, ESP provides an effective and scalable solution for dataset condensation, resulting in the synthesis of highly informative compact datasets. This technique enables the application of modern deep learning approaches in resource-constrained settings, where memory or computational resources are limited. Moreover, ESP contributes to minimizing the energy consumption needed for training models.

## 6. Limitations and Future Work

Despite the effectiveness of our Expert Subspace Projection (ESP) approach in reducing memory usage and computational requirements compared to the previous trajectory matching [10] approach, it is crucial to acknowledge that ESP still operates within a bi-level optimization framework. Consequently, extending ESP to large datasets that contain high-resolution images presents a significant challenge, similar to previous bi-level optimization methods [1,6,7,10,36,37]. This limitation hampers the application of dataset condensation to tasks such as fine-grained classification, which heavily depends on high-resolution images for capturing intricate details. Therefore, it is imperative to focus further efforts on exploring strategies to minimize memory usage and computational requirements. Promising directions include disentangling the outer optimization from the inner optimization and approximating the inner optimization using a convex proxy model.

## Figures and Tables

**Table 1:** **Classification accuracies (%) on ConvNet-3.** The results of other methods are reported in their respective papers [1,5,6,7,10,36,37,38]. For our method, we report mean accuracy and standard deviation over five runs with different random seeds.

Dataset	SVHN			CIFAR10			CIFAR100		TinyImageNet	
**Metric**	**Accuracy (%)**			**Accuracy (%)**			**Accuracy (%)**		**Accuracy (%)**	
**Images/Class**	**1**	**10**	**50**	**1**	**10**	**50**	**1**	**10**	**1**	**10**
**Param./Class**	**3072**	**30,720**	**153,600**	**3072**	**30,720**	**153,600**	**3072**	**30,720**	**12,288**	**122,880**
Random	14.6_±1.6_	35.1_±4.1_	70.9_±0.9_	14.4_±2.0_	26.0_±1.2_	43.4_±1.0_	4.2_±0.3_	14.6_±0.5_	1.4_±0.1_	5.0_±0.2_
Herding	20.9_±1.3_	50.5_±3.3_	72.6_±0.8_	21.5_±1.2_	31.6_±0.7_	40.4_±0.6_	8.4_±0.3_	17.3_±0.3_	2.8_±0.2_	6.3_±0.2_
DC [6]	31.2_±1.4_	76.1_±0.6_	82.3_±0.3_	28.3_±0.5_	44.9_±0.5_	53.9_±0.5_	12.8_±0.3_	25.2_±0.3_	-	-
DSA [7]	27.5_±1.4_	79.2_±0.5_	84.4_±0.4_	28.8_±0.7_	52.1_±0.5_	60.6_±0.5_	13.9_±0.3_	32.3_±0.3_	-	-
DM [5]	20.3_±2.1_	73.5_±1.0_	84.2_±0.0_	26.0_±0.8_	48.9_±0.6_	63.0_±0.4_	11.4_±0.3_	29.7_±0.3_	3.9_±0.2_	12.9_±0.4_
KIP to NN [36]	57.3_±0.1_	75.0_±0.1_	80.5_±0.1_	49.9_±0.2_	62.7_±0.3_	68.6_±0.2_	15.7_±0.2_	28.3_±0.1_	-	-
CAFE + DSA [37]	42.9_±3.0_	77.9_±0.6_	82.3_±0.4_	31.6_±0.8_	50.9_±0.5_	62.3_±0.4_	14.0_±0.3_	31.5_±0.2_	-	-
Traj. Matching [10]	-	-	-	46.3_±0.8_	65.3_±0.7_	71.6_±0.2_	24.3_±0.3_	40.1_±0.4_	8.8_±0.3_	23.2_±0.2_
IDC [38]	68.1_±0.1_	87.3_±0.2_	90.2_±0.1_	50.0_±0.4_	67.5_±0.5_	74.5_±0.1_	-	44.8_±0.2_	-	-
KFS [1]	82.9_±0.4_	91.4_±0.2_	92.2_±0.1_	59.8_±0.5_	72.0_±0.3_	75.0_±0.2_	40.0_±0.5_	**50.6** **_±0.2_**	22.7_±0.2_	**27.8** **_±0.2_**
**ESP (ours)**	**84.8** **_±0.3_**	**91.6** **_±0.1_**	**92.8** **_±0.1_**	**63.0** **_±0.4_**	**73.8** **_±0.2_**	**76.1** **_±0.3_**	**41.1** **_±0.3_**	48.0_±0.1_	**24.9** **_±0.3_**	26.6_±0.5_
Full dataset	95.4_±0.1_			84.8_±0.1_			56.2_±0.3_		37.6_±0.4_	

**Table 2:** **Cross-architecture experiments.** Conv3, RN10, and DN121 denote ConvNet-3, ResNet-10, and DenseNet-121, respectively. We train on ConvNet-3 and evaluate on the three architectures. The results of other methods are reported in their respective papers [1,5,7,38]. For our method, we report mean accuracy and standard deviation over five runs with different random seeds.

Dataset	Images/Class	1			10			50		
	**Metric**	**Accuracy (%)**			**Accuracy (%)**			**Accuracy (%)**		
	**Test Architecture**	**Conv3**	**RN10**	**DN121**	**Conv3**	**RN10**	**DN121**	**Conv3**	**RN10**	**DN121**
SVHN	DSA [7]	27.5_±1.4_	13.2_±1.1_	13.3_±1.4_	79.2_±0.5_	19.5_±1.5_	23.1_±1.9_	84.4_±0.4_	41.6_±2.1_	58.0_±3.1_
	DM [5]	20.3_±2.1_	10.5_±2.8_	13.6_±1.0_	73.5_±1.0_	28.2_±1.5_	24.8_±2.5_	84.2_±0.0_	54.7_±1.3_	58.4_±2.7_
	IDC [38]	68.1_±0.1_	39.6_±1.5_	39.9_±2.9_	87.3_±0.2_	83.3_±0.2_	82.8_±0.2_	90.2_±0.1_	89.1_±0.2_	91.0_±0.3_
	KFS [1]	82.9_±0.4_	75.7_±0.8_	81.0_±0.7_	91.4_±0.2_	90.3_±0.2_	89.7_±0.2_	92.2_±0.1_	90.9_±0.2_	90.2_±0.2_
	**ESP (ours)**	**84.8** **_±0.3_**	**84.7** **_±0.6_**	**82.0** **_±0.1_**	**91.6** **_±0.2_**	**93.5** **_±0.1_**	**90.7** **_±0.3_**	**92.8** **_±0.1_**	**93.7** **_±0.1_**	**91.0** **_±0.5_**
	Full dataset	95.4_±0.1_	93.8_±0.5_	89.1_±0.8_	95.4_±0.1_	93.8_±0.5_	89.1_±0.8_	95.4_±0.1_	93.8_±0.5_	89.1_±0.8_
CIFAR10	DSA [7]	28.8_±0.7_	25.1_±0.8_	25.9_±1.8_	52.1_±0.5_	31.4_±0.9_	32.9_±1.0_	60.6_±0.5_	49.0_±0.7_	53.4_±0.8_
	DM [5]	26.0_±0.8_	13.7_±1.6_	12.9_±1.8_	48.9_±0.6_	31.7_±1.1_	32.2_±0.8_	63.0_±0.4_	49.1_±0.7_	53.7_±0.7_
	IDC [38]	50.0_±0.4_	41.9_±0.6_	39.8_±1.2_	67.5_±0.5_	63.5_±0.1_	61.6_±0.6_	74.5_±0.1_	72.4_±0.5_	71.8_±0.6_
	KFS [1]	59.8_±0.5_	47.0_±0.8_	49.5_±1.3_	72.0_±0.3_	70.3_±0.3_	69.2_±0.4_	75.0_±0.2_	75.1_±0.3_	**76.3** **_±0.4_**
	**ESP (ours)**	**63.0** **_±0.4_**	**50.4** **_±0.5_**	**51.6** **_±0.6_**	**73.0** **_±0.3_**	**71.8** **_±0.4_**	**69.4** **_±0.3_**	**75.9** **_±0.3_**	**75.3** **_±0.2_**	73.0_±0.5_
	Full dataset	84.8_±0.1_	87.9_±0.2_	90.5_±0.3_	84.8_±0.1_	87.9_±0.2_	90.5_±0.3_	84.8_±0.1_	87.9_±0.2_	90.5_±0.3_

**Table 3:** **Cross-architecture experiments on EfficientNet.** ENs denotes EfficientNetv2-s [39]. We train on ConvNet-3 and evaluate on EfficientNetv2-s. The reported results are solely based on our own experiments.

Dataset	Images/Class	1	10	50
	**Metric**	**Accuracy (%)**	**Accuracy (%)**	**Accuracy (%)**
	**Test Architecture**	**ENs**	**ENs**	**ENs**
CIFAR10	DSA [7]	16.5_±0.3_	25.6_±0.4_	33.7_±0.2_
	DC [6]	16.1_±1.7_	22.3_±1.4_	25.7_±0.9_
	Traj. Matching [10]	17.7_±0.2_	24.0_±0.4_	33.9_±0.6_
	**ESP (ours)**	**35.3** **_±0.4_**	**55.0** **_±1.2_**	**63.9** **_±0.8_**
	Full dataset	98.7_±0.2_	98.7_±0.2_	98.7_±0.2_

**Table 4:** Classification accuracies (%) on CIFAR10 [24]. We maintain the other hyper-parameters and only change the loss.

Loss	$L_{\mathrm{Proj}}$	$L_{\mathrm{DM}}$	Images/Class		
			**1**	**10**	**50**
Proj	✓		41.3_±0.2_	40.6_±0.4_	39.5_±0.3_
DM		✓	48.0_±0.3_	71.3_±0.3_	74.0_±0.1_
Proj + DM	✓	✓	**62.6** **_±0.1_**	**73.0** **_±0.3_**	**75.9** **_±0.2_**

## Data Availability

The data presented in this study are all openly available. CIFAR10/100 is found at https://www.cs.toronto.edu/kriz/cifar.html, accessed on 11 August 2023; SVHN is found at http://ufldl.stanford.edu/housenumbers/, accessed on 11 August 2023; and TinyImageNet is found at https://www.kaggle.com/c/tiny-imagenet or https://www.image-net.org/download, accessed on 11 August 2023.
